# Supplementary material for: Risk factors for community-acquired respiratory infections in a non-pandemic context: Secondary analysis of the PRIMIT study
Source: PLoS One. 2022 Nov 17;17(11):e0277201. doi: 10.1371/journal.pone.0277201 (PMC9671441; doi:10.1371/journal.pone.0277201)
Supplement: Attachment — Submitted filename: Response to Reviewers.docx [file pone.0277201.s001.docx]

|  |  |  |  | |
| --- | --- | --- | --- | --- |
|  |  | | | |
|  |  | | **centre for ACADEMIC PRIMARY CARE**  NIHR School of Primary Care Research  Bristol Medical School  Population Health Sciences  Canynge Hall, 39 Whatley Road  Bristol, BS8 2PS | |
|  |  |  | Ashley Hammond  Research Fellow  ashley.hammond@bristol.ac.uk  19 August 2022 | |
| Dear Lorena, | | | |  |

**Re:** **Risk factors for community-acquired respiratory infections in a non-pandemic context: secondary analysis of the PRIMIT study (PONE-D-21-30671)**

Many thanks for your 23 June 2022 email inviting us to revise our manuscript. As requested, two versions are submitted (‘Revised Manuscript with Track Changes’ and ‘Manuscript’).

The table below describes our responses to both journal and reviewer comments, and the changes made to the manuscript where necessary.

We were also asked to provide an updated Funding Statement, we would like this updated to “PRIMIT was funded by the Efficacy and Mechanism Evaluation Programme (09/800/22), in partnership between the MRC and NIHR. ADH is supported by a NIHR Senior Investigator Award (NIHR 200151), which funded this secondary data analysis. The views expressed in this publication are those of the author(s) and not necessarily those of the MRC, NHS, NIHR, or the Department of Health and Social Care.”

Yours sincerely,

Ashley Hammond

# PONE-D-21-30671 Risk factors for community-acquired respiratory infections in a non-pandemic context: secondary analysis of the PRIMIT study

|  | **Comments** | **Author responses** | **Changes to manuscript** |
| --- | --- | --- | --- |
|  | **Journal Comments** | |  |
| 1. | Please ensure that your manuscript meets PLOS ONE's style requirements, including those for file naming. | We have now reformatted all headings, subheadings and text in line with PLoS One formatting and style requirements. | Reformatted throughout Manuscript. |
| 2. | Please add the registration number of the original study in the Method section of your manuscript. | We have now added the study registration number of the original study to the methods. | Text added to lines 77-78. |
| 3. | We note that the grant information you provided in the ‘Funding Information’ and ‘Financial Disclosure’ sections do not match. | We have updated our ‘Financial Disclosure’ section so that it matches our ‘Funding Information’ statement, detailed in our response below in comment 4. | Financial Disclosure section updated to match our Funding Statement. |
| 4. | Thank you for stating the following in the Acknowledgments Section of your manuscript:  “PRIMIT was funded by the Efficacy and Mechanism Evaluation Programme (09/800/22), in partnership between the MRC and NIHR. ADH is supported by a NIHR Senior Investigator Award (NIHR 200151). The views expressed in this publication are those of the author(s) and not necessarily those of the MRC, NHS, NIHR, or the Department of Health and Social Care.”  We note that you have provided additional information within the Acknowledgements Section that is not currently declared in your Funding Statement. Please note that funding information should not appear in the Acknowledgments section or other areas of your manuscript. We will only publish funding information present in the Funding Statement section of the online submission form.  Please remove any funding-related text from the manuscript and let us know how you would like to update your Funding Statement. Currently, your Funding Statement reads as follows:  “PRIMIT was funded by the Efficacy and Mechanism Evaluation Programme (09/800/22), in partnership between the MRC and NIHR. ADH is supported by a NIHR Senior Investigator Award (NIHR 200151). The views expressed in this publication are those of the author(s) and not necessarily those of the MRC, NHS, NIHR, or the Department of Health and Social Care.”  Please include your amended statements within your cover letter; we will change the online submission form on your behalf. | We have removed the acknowledgements section in our Manuscript, as this reflects funding information only.  We would like to update our funding statement as below, and have identified this in our covering letter:  PRIMIT was funded by the Efficacy and Mechanism Evaluation Programme (09/800/22), in partnership between the MRC and NIHR. ADH is supported by a NIHR Senior Investigator Award (NIHR 200151), which funded this secondary data analysis. The views expressed in this publication are those of the author(s) and not necessarily those of the MRC, NHS, NIHR, or the Department of Health and Social Care. | Acknowledgements section removed.  Funding text removed on lines 76-77.  Updated Funding Statement detailed in our cover letter. |
|  | **Reviewer’s Comments** | | |
| 5. | Abstract can be rewritten using the following subsections: (a) Objectives, (b) Methods, (c) Results and (d) Conclusions | We have now reformatted the abstract in line with PLoS One guidance, using the headings suggested by the reviewer. | Abstract reformatted on Page 2-3. |
| 6. | Results were generated from a study completed in 2013; not sure what knowledge gain we may have in 2022! | We thank the reviewer for their comment. We feel that (aside from the COVID-19 pandemic), the nature of the relationship between risk factors and community-acquired RTI doesn’t seem likely to be time-dependent. Thus, although the data from the PRIMIT study are from 2013, there is no reason why it may not still be informative.  We (AH and ADH) recently published a systematic review (Hammond et al, BMC Infectious Diseases, 2021) which also highlighted that we still know little about risk factors for community-acquired RTIs. Therefore we believe this secondary analysis of a randomised trial will still provide important new findings.    The nature of the COVID-19 pandemic and its impact on handwashing could cause a shift in this perspective however, and is something we feel should be highlighted in our discussion. We have added some additional text to our manuscript. | Additional text added to lines 208-212. |
| 7. | Outcomes: Primary outcome definition is wrong! Maybe the authors meant that the binary outcome means 1 if participants reported >= 1 RTI at 16 weeks; 0 otherwise? | We have re-worded our text describing the primary outcome to clarify. | Text re-worded on lines 81-83. |
| 8. | Dataset has a decent number of intervention & control participants, and collected from 344 primary care centres. So, why not the centre effect was adjusted for via a GEE-type approach, or a random intercept logistic regression model? | Clustering at the centre level is considered non-ignorable, and therefore something that needs to be taken into account as the reviewer suggests, if two conditions are met: the ICC must be non-zero and the correlation of patient assignments within the cluster must be non-zero. This has previously been assessed in the PRIMIT trial (<https://bmcmedresmethodol.biomedcentral.com/articles/10.1186/s12874-020-00971-7>) and clustering was deemed ignorable. The ICC was 0.009 and the allocation of patients used simple randomisation. Therefore a GEE or random intercept approach would be unlikely to add to the interpretation but with such a large number of clusters might cause convergence issues. | No change. |
| 9. | Logistic regression Goodness-of-fit, say via Hosmer-Lemeshow statistics, were not presented. | The evaluation of goodness of fit in logistic regression is challenging. Tests such as Hosmer-Lemeshow are highly influenced by sample size, which is large here and therefore likely to lead to a rejection of the null hypothesis even if model fit is adequate. Moreover, the purpose of this analysis was not to select the best model but to include all variables on which data had been collected and explore which were independently associated with the outcome. We have tried to make it clear in our limitations there is data we would have liked to collect that might have improved model fit but this was not possible in a large RCT with multiple follow up points due to participant burden. | No change. |
| 10. | I see that this simple yet rigorous manuscript has adequately explained the whole idea of restriction of social engagements could be protective in a sense of risk of contracting RTI. The clinical criteria for its subject may seem to be too permissive, though; e.g. there is no explanation of the severity of the RTI (new, recurrent, acute, an acute episode of a chronic, or debilitating RTI), there is no description of the location of RTI (upper, lower, or mixed), and there is no description of the duration of the RTI, as there are many kinds of RTI which has different outcome. This should be addressed in the manuscript, at least in a brief text. | We agree with the reviewer that this should be mentioned as a limitation of the study. We have added additional text to our discussion section. | Additional text added on lines 156-159. |
| 11. | This manuscript is a secondary analysis of the PRIMIT Study and shows risk factors associated with the incidence of RTIs. This is well written and illustrated paper which presents the results from a large self-reported survey, and the relationship between acquisition of RTIs and risk factors described is more straightforward. | The reviewer is positive here but this isn’t a survey. These are questionnaire responses from a randomised trial. | No change. |
| 12. | Regarding the results of "education after the age of 10" (aOR 1.09, 95% CI 1.02 to 1.17, p=0.01) shown in Table 2, the text states that years of education do not affect the acquisition of RTI, but it is necessary to discuss this issue further. | We agree with the reviewer that we have mistakenly overlooked this finding. We have adjusted our text to acknowledge this finding. We agree that possible explanations should be offered for this finding and have also added additional text to our discussion section. | Additional text on lines 26-27, 118-119, 142-143, 170-174, 234. |
| 13. | Regarding the point that older adults are less likely to acquire RTIs, as noted in the text, there is a discrepancy with the findings in the previous literature. The authors should discuss this point. | We have addressed this discrepancy in our discussion in lines 155-158. | No change. |
| 14. | The definition of “skin condition that impacted handwashing” should be described in the text, because it is one of the important findings in this paper. | This question was self-defined by the participant. They were asked whether they had any skin condition that impacted their handwashing, rather than there being a specific definition of what that condition might be. We feel that this should be described more clearly, so we have added in some additional text into our discussion section. | Additional text added on lines 159-165. |
